# Students’ and Examiners’ Experiences of Their First Virtual Pharmacy Objective Structured Clinical Examination (OSCE) in Australia during the COVID-19 Pandemic

**DOI:** 10.3390/healthcare10020328

**Published:** 2022-02-09

**Authors:** Vivienne Mak, Sunanthiny Krishnan, Sara Chuang

**Affiliations:** 1 Faculty of Pharmacy and Pharmaceutical Sciences, Monash University, Parkville, VIC 3052, Australia; sara.chuang@monash.edu; 2 School of Pharmacy, Monash University Malaysia, Bandar Sunway, Subang Jaya 47500, Malaysia; Sunanthiny.S.Krishnan@monash.edu

**Keywords:** e-learning, healthcare education, telehealth, OSCE, pharmacy, communication

## Abstract

Objective Structured Clinical Examinations (OSCEs) are routinely used in healthcare education programs. Traditionally, students undertake OSCEs as face-to-face interactions to assess competency in soft skills. Due to physical distancing restrictions during COVID-19, alternative methods were required. This study utilized a mixed-method design (online survey and interviews) to evaluate second-year pharmacy students’ and examiners’ experiences of their first virtual OSCEs in Australia. A total of 196 students completed their first virtual OSCE in June 2020 of which 190 students completed the online survey. However, out of the 190 students, only 88% (*n* = 167) consented to the use of the data from their online survey. A further 10 students and 12 examiners were interviewed. Fifty-five students (33%) who participated in the online survey strongly agreed or agreed that they preferred the virtual experience to face-to-face OSCEs while 44% (*n* = 73) neither agreed nor disagreed. Only 20% (*n* = 33) felt more anxious with the virtual OSCEs. Additionally, thematic analysis found non-verbal communication as a barrier during the OSCE. Positive aspects about virtual OSCEs included flexibility, decreased levels of anxiety and relevance with emerging telehealth practice. The need for remote online delivery of assessments saw innovative ways of undertaking OSCEs and an opportunity to mimic telehealth. While students and examiners embraced the virtual OSCE process, face-to-face OSCEs were still considered important and irreplaceable. Future opportunities for OSCEs to be delivered both face-to-face and virtually should be considered.

## 1. Introduction

Objective Structured Clinical Examinations (OSCEs) have been used routinely in healthcare education programs. Traditionally, students undertake OSCEs as face-to-face interactions to assess competency in soft skills including problem solving, empathy and communication. Students move through a circuit of purposely designed stations to assess them in a standardized simulated environment. At each station, students complete a clinical task within a predetermined time in the presence of a simulated patient. These simulated patients adhere to a strict script to ensure that each student is assessed in a standardized manner. In the pharmacy course, an OSCE is used to evaluate students simulating a patient–pharmacist encounter in a community or hospital pharmacy setting. There may also be instances where the student will simulate a pharmacist encounter with another healthcare professional. These OSCEs assess content knowledge as well as problem solving, communication skills and empathy. OSCEs are considered the gold standard in assessing skills development in clinical students due to their objectivity and reliability [1].

Due to physical distancing restrictions during the COVID-19 pandemic, alternative methods of conducting OSCEs were required as face-to-face OSCEs were no longer possible at many institutions. Fortunately, due to the advances in technology and the rising demand for telehealth solutions, conversion of OSCEs to virtual or online formats occurred quickly. Utilizing telecommunication platforms such as Zoom, traditional face-to-face OSCE scenarios were adapted for online delivery. This was proven to be done successfully in other health professions such as in a medical faculty [2]. There are many considerations before conducting a virtual OSCE, including ensuring that both examiners and students have the appropriate resources, including a functional device compatible with Zoom, a video camera, audio capabilities, and a good and stable internet connection. This reduces any potential for technological challenges that may occur during the OSCE. As this was the first time pharmacy students and examiners were undertaking their OSCEs in a virtual format, it was important to provide clear instructions and training on how the virtual OSCEs would be conducted [3].

While this swift adaptation to virtual OSCEs afforded reasonable continuity of the assessment, there were considerable challenges with this mode of examination that course coordinators had to navigate in order to achieve the learning outcomes of these performance-based competency assessments. In particular, it is difficult to capture the full range of non-verbal communication skills on synchronous video conferencing media, due to audiovisual limitations such as screen size, position of the student on screen as well as potential poor video quality [4,5].

Poor internet connectivity further impedes the smooth process of “live interactive” assessments. Low bandwidth connections, especially among students living in rural areas with limited internet access, could be particularly challenging and stressful during time-sensitive assessments such as OSCEs. There have been previous reports that poor internet access could be a potential for concern around equity in assessments [6,7]. In a survey conducted among 307 agricultural graduates from different universities in India, a lack of internet connectivity was reported as the major hindrance to online learning, more acutely affecting those from remote areas [7]. Technology and connection issues are of concern regardless of location. In reporting the outcome of a virtual teaching OSCE (vTOSCE) with pharmacy students at Ferris State University, the authors noted that both students as well as faculty members experienced connectivity issues during the vTOSCE [8]. Similarly, another study reported that many students experienced internet connectivity issues during their first remote OSCE in the United States, thus further highlighting the global nature of the problem [9].

Furthermore, academic integrity is also a serious cause for concern with virtual OSCEs as non-invigilated remote assessments are subject to cheating and collusion among students [6,10]. Many studies have shown that students in unproctored online assessments perform better compared to those in proctored environments [11,12,13]. Notably, students who undertake the OSCE at a later examination schedule may have an advantage due to the potential for students who have completed their examination prior to share exam information with others who have not [14]. Updike et al. (2021) reported an incident of a possible breach of OSCE integrity in a high-stakes assessment where students could potentially have taken screenshots or photos of electronically shared OSCE case materials [15]. The authors have suggested various approaches, including creating different case scenarios and reducing administration time during examinations to limit discussions among students and to protect the integrity of the virtual OSCEs [15].

Given these challenges, the conversion from conventional face-to-face to virtual OSCEs requires varying degrees of modifications to the assessment format, as the former is not entirely replicable virtually. Several papers described the functional processes of adaptation and the changes necessitated by the online delivery. Some of these amendments include fewer OSCE stations, conversion of interactive stations to passive stations, written stations examinable via the institution’s Learning Management System (LMS), changes to testing procedures using proctoring software (e.g., ExamSoft), video recording of the examination as well as recruiting internal faculty members as simulated patients instead [1,15,16]. There have been many innovative OSCE conversions during the pandemic, including one in the United States where students were “advised to recruit an adult within their residence to be their patient” [17] (p. 247) as part of a physical examination on a simulated patient. Evidently, many modifications were essentially driven by the logistical complexity of implementing “live interactive” online assessments and their feasibility during the pandemic.

At the Faculty of Pharmacy and Pharmaceutical Sciences, Monash University, second-year pharmacy students completed an OSCE in June 2020. This OSCE consisted of two stations situated within the community pharmacy setting. The examination was designed to assess students’ skills in problem solving, oral communication and empathy as a pharmacist, using role play with a simulated patient suffering from asthma or eczema. As with other faculties during the pandemic, the options were to either delay the OSCE until lockdown restrictions were lifted or to continue the assessment virtually. Given that there was a closure on international borders, shutdown of non-essential services and movement restrictions in Melbourne, Australia [18], we decided to convert the OSCE to an online format and utilized the Zoom video conferencing platform as the medium for the assessment. Students were then able to be assessed synchronously by examiners who also played the role of the patients.

Whilst students had previously experienced OSCEs via face-to-face delivery in the first year of their pharmacy degree, this was their first exposure to the online format. To prepare faculty and students for the online format of the OSCE, training was conducted online. Students practised and were formatively assessed on their role plays in weekly workshops conducted over Zoom with a pharmacist facilitator. They were also provided an OSCE overview video that showcased the process and what to expect in a virtual OSCE. Faculty completed an online training module that consisted of the virtual OSCE process, how to assess online and the case content.

There have been many international reports describing the experiences of undertaking a virtual OSCE during the pandemic; however, this is an Australian study that adds to the literature on pharmacy students’ and examiners’ experiences of their first virtual OSCE utilizing a mixed-method study design. The aim of our study was to evaluate pharmacy students’ and OSCE examiners’ experiences of their first virtual OSCE.

The research questions for this study were as follows:

1. What were the pharmacy students’ experience of undertaking a virtual OSCE?
2. What were the examiners’ experience of the virtual OSCEs?
3. Were there differences in experiences between the virtual OSCEs and face-to-face OSCEs?

## 2. Materials and Methods

### 2.1. Virtual OSCE Process

The virtual OSCE process involved five steps, as summarized in Table 1 [3]. A day prior to the OSCE, the examination times were published to the students. The published times were given only a day prior to the OSCE to minimize any opportunities for collusion. We also had a Zoom license that allowed for administrators to host concurrent meetings and be dialled into multiple Zoom Meetings at the same time.

On the day of the OSCE, the students dialled into the Zoom Meeting using the link provided to them at their published time. The Zoom Meeting was referred to as the “Zoom OSCE Lobby”. Once they dialled into the Zoom Meeting, they immediately appeared in a Zoom “waiting room”. Students waited until the administrators admitted them into the Zoom OSCE Lobby. If there were delays, the administrators communicated with those in the waiting room using the Zoom chat function.

Once admitted into the Zoom OSCE Lobby, an administrator would check their identifications and also test that their internet connection was stable, as well as their audio and visual capabilities were sufficient for the examination. This was to determine if there was obvious lag in the student’s internet connection or if there was any problem with their audio and video. In the event that there were issues, a member of staff assisted the student virtually to overcome the technical difficulties. The students were then given a quick briefing of the processes for the day.

When the identification process and technical set up was completed, the students were then sent to their Zoom OSCE Room. This was a new Zoom Meeting link where the examiner was located. An administrator provided this Zoom Meeting link to the student via a private chat. This administrator was dialled into the Zoom OSCE Lobby and also the Zoom OSCE Room. This meant that once the student clicked the Zoom OSCE Room link, the administrator was able to observe the student transporting themselves virtually to their exam room.

Once the student arrived in the Zoom OSCE Room, they automatically appeared in another Zoom waiting room. The students were briefed to wait patiently until the examiner admitted them into the exam room. This often occurred either immediately or up to 5 min post arrival. Once admitted into the Zoom OSCE Room, the examiner would ask for their full name and student ID number to confirm that they had the correct student. The examiner then provided a quick introduction to the exam and informed the student once the timer began. In this OSCE, the timer was set for seven minutes and the examiner was also simulating the patient. Marking was conducted on Qualtrics and a link was provided to the examiners prior to the commencement of the assessment. At the end of the seven minutes, the examiner would end the interaction and the students were asked to leave the Zoom OSCE Room. The examiner then completed their marking and repeated the process by admitting the next student from the Zoom waiting room.

### 2.2. Study Design

This study employed a mixed-method design. An online survey via Qualtrics and semi-structured interviews were conducted.

At Monash University, after each OSCE, students complete a post-OSCE reflection online survey where they reflect and rate their own performance. As part of this usual online survey, additional questions were included to explore their experiences of their first virtual OSCE for this study. These questions were developed by the course instructors and evaluated for face validity. These questions included statements ranked using the 5-point Likert scale, as well as opportunities for open-ended comments to elaborate on their answers pertaining to the virtual OSCE:

(a) I prefer virtual OSCEs compared to face-to-face OSCEs
  (b) Please elaborate on the reasons for your answer
(a) I felt more anxious during the virtual OSCE compared to face-to-face OSCE
  (b) Please elaborate on the reasons for your answer
(a) The virtual OSCE felt more challenging compared to a face-to-face OSCE
  (b) Please elaborate on the reasons for your answer

Complete the following:

4. The best thing about the virtual OSCE is…
5. If I could improve something about the virtual OSCE, I would…
6. Please provide final comments (if any) about the virtual OSCE

As part of this online survey, students were invited to consent to a follow-up semi-structured interview to further explore their experiences. All examiners received an invitation to participate in a semi-structured interview to discuss the benefits and disadvantages of virtual OSCEs, as well as how they conducted the virtual OSCE as an examiner.

### 2.3. Data Collection and Analysis

The OSCE was conducted in June 2020. The online survey was administered to 196 second-year pharmacy students immediately after completion of their first virtual OSCE. All students completed the survey as part of the routine quality improvement process for the course but were required to consent to the use of their data for this study. The quantitative online survey data were analyzed descriptively.

Students who completed the online survey were also invited to participate in a further interview. A total of 25 students indicated that they were interested in being interviewed. All 18 OSCE examiners who examined in the OSCE were invited to participate in an interview.

All interviews were conducted via Zoom Meetings and transcribed verbatim using a speech to text transcription service called Otter.ai. All open-ended comments in the online survey and interviews were included in the qualitative data analysis. Qualitative data were thematically analyzed, where open-ended comments from the online survey and interview transcripts were read repeatedly for initial familiarization and coded. The codes were then grouped into themes and discussed with the project team until consensus was reached.

## 3. Results

A total of 190 students (96.9%) completed the online survey; however, only 87.9% (*n* = 167) of those who completed the online survey consented to the use of their survey data.

Ten of the 25 students who indicated their interest to be interviewed were contacted and interviews arranged. A total of 12 examiners agreed to be interviewed.

### 3.1. Quantitative Data

The survey results showed that 33% of students strongly agreed or agreed that they preferred the virtual OSCE experience to face-to-face OSCE while 44% showed no preference to either methods. Only 20% felt more anxious compared to the face-to-face OSCE while 12% agreed that the virtual OSCE felt more challenging (Table 2).

### 3.2. Qualitative Themes

There were five themes that arose from the open-ended survey comments from students and the interviews with both students and examiners. Positive aspects about virtual OSCEs included flexibility, decreased levels of anxiety and the relevance with emerging practice such as telehealth. Many students also indicated that they practiced with their peers online in preparation for the virtual OSCEs. Non-verbal communication was seen to be challenging virtually.

#### 3.2.1. Theme 1: Flexibility, Convenience and Comfort

One of the main themes in this study was the increased flexibility and convenience of the virtual OSCEs. There were several students and examiners who expressed that the opportunity to undertake the examinations at home was seen as a benefit (Table 3, Subtheme 1.1).

A student further described that they felt less fatigued due to not needing to travel to university and that they felt that it was a better use of their time. The ability to undertake the OSCEs in the comfort of their own home was also seen to be a preference in a number of students (Table 3, Subtheme 1.2).

Examiners on the other hand also found it convenient conducting virtual OSCEs as they were able to have the examination materials they require on their screens/monitors. This is unlike a face-to-face examination where they had to either memorize the materials or assess the student after the interaction. Additionally, they were able to have the comfort of setting up a backup plan at home in case of technical difficulties. As with examiners and their convenience in materials, students also expressed the convenience in their ability to access their notes and resources from home (Table 3, Subtheme 1.3).

Nevertheless, even though students were in the comfort of their own home, technical difficulties and challenges were still possible. One student identified that the inability to see the physical examiner meant that they were questioning if their technology was working before the examination while in the Zoom waiting room. Another student indicated that even in the convenience of their home, the internet connection could still be an issue (Table 3, Subtheme 1.4).

As OSCEs are time-sensitive assessments, where each station needs to commence at a specific time, the examiners felt that there was flexibility in when the student commences the interaction. Examiners had to admit the students from the Zoom waiting room and thus had control of the assessment times to some extent (Table 3, Subtheme 1.5).

Students were not only thinking about flexibility and convenience for their own personal examinations. A couple of students were also expressing flexibility and convenience for their future practice (Table 3, Subtheme 1.6).

#### 3.2.2. Theme 2: Decreased Anxiety and Nerves

A number of students expressed that doing the OSCE virtually made them feel secure as they felt that they were in a safe environment. Interestingly, they expressed that not being able to speak to their peers or be around other nervous students helped them stay calm and mentally prepared for the assessment (Table 4, Subtheme 2.1).

Examiners also noticed that students appeared less nervous during the virtual OSCEs compared to face-to-face OSCEs. Both students and examiners felt that having set up their surroundings to be familiar to them, including setting up their own timer for the OSCE and having their own ways of preparing themselves, helped them with their nerves; they were less stressful (Table 4, Subtheme 2.2).

#### 3.2.3. Theme 3: Skill Development and Future Training for Telehealth

Many students saw this first opportunity to undertake virtual OSCEs as good training for their future as pharmacists and with the evolving roles with the use of technology and digital health (Table 5, Subtheme 3.1).

They saw the pandemic to have allowed for this opportunity to keep up with practice considering that uptake to telehealth was accelerated during COVID-19. In addition, it was considered to be a flexible way of working in the future. Examiners’ views were also consistent with how students viewed the future of telehealth and felt that this was a great way to train students (Table 5, Subtheme 3.2).

Nevertheless, examiners were concerned about the training of pharmacy students solely on the use of virtual OSCEs. They felt that a mix of both virtual and face-to-face OSCEs would be most beneficial. Similarly, students expressed that they would ideally like to have a combination of face-to-face and virtual OSCEs (Table 5, Subtheme 3.3).

#### 3.2.4. Theme 4: Non-Verbal Communication a Barrier

Although there were many positives with the virtual OSCEs, both examiners and students identified non-verbal communication as the main barrier during the OSCE. Students expressed challenges in displaying good non-verbal skills while examiners found difficulty evaluating these skills virtually (Table 6, Subtheme 4.1). Empathy was highlighted as a skill that was difficult to assess virtually from an examiner’s perspective. Students also expressed difficulty in picking up empathy cues from simulated patients (Table 6, Subtheme 4.2).

#### 3.2.5. Theme 5: Practice in Preparation for Virtual OSCE

Many students also said that they practised online before the virtual OSCEs to be more familiar with the technology and also to mimic the style of assessments they were going to undertake (Table 7).

## 4. Discussion

This study found that it was still feasible to conduct OSCEs virtually and both examiners and students felt that this alternative assessment method had many positive aspects compared to face-to-face OSCEs. Nevertheless, there was still a preference for traditional methods and a desire for a hybrid version with both virtual and face-to-face OSCEs in the future. A major benefit of virtual OSCEs felt by students was a decrease in anxiety and nerves for the assessment. Students who were interviewed all indicated less anxiety during virtual OSCEs, which was consistent with the survey results, where only 20% of the students agreed that they felt more anxious during the virtual OSCEs compared to face-to-face OSCEs. Studies in other countries also found that students were less anxious in online OSCEs [19,20], while traditional face-to-face OSCEs have always been intimidating and stressful [21]. The decreased stress levels expressed by the students in our study could potentially be due to a result of being in the comfort of their home surroundings and, in some cases, the absence of peer anxiety. However, it should be noted that a recent systematic review examining the relationship between OSCE-associated test anxiety and OSCE performance found that there was little influence on student performance [22]. Another study compared the performance of two cohorts of pharmacy students with one cohort in 2020 that undertook the OSCE virtually during the pandemic and the second cohort in 2019 with traditional methods [23]. The authors found that students who undertook their OSCE virtually performed as well as those who completed it in-person via traditional methods [23].

An emerging theme in our study was the increased flexibility of virtual OSCEs expressed by both students and examiners. The reduced travel time and the ability to complete the OSCE from almost any location appealed strongly to all participants. Given the situation during the pandemic whereby students were potentially located overseas due to the closure of international borders, this flexibility to conduct the examinations online was essential to prevent delays in students’ course progressions. A study found that 60% of their academic staff members felt that working from home was more flexible than traditional methods during COVID-19 [24]. Flexibility to work from anywhere assisted faculty members especially if there was a need to manage family responsibilities [25]. This was particularly useful for faculty members with children that require home schooling during the pandemic. Virtual OSCEs also provided greater ease in logistical and spatial requirements, which is often a limiting factor to face-to-face OSCEs [26]. Nevertheless, our virtual OSCE was conducted with multiple Zoom Meetings hosted concurrently. This could be limited if other educators decide to replicate similar methods of conducting the virtual OSCEs as special scheduling privileges are required for administrators to host multiple concurrent Zoom Meetings and can cost more to institutions [27].

There was also recognition of virtual OSCE by our student participants as an avenue to develop skills in the direction of telehealth. Telehealth has been steadily redesigning the landscape of healthcare systems since the rapid advancements and utilization of telecommunication media over the past two decades, which was accelerated due to the COVID-19 pandemic [28]. Originally conceptualized to overcome barriers to healthcare access, particularly geographical distance and restricted mobility [28,29], the value of telemedicine has expanded to reduce waiting times, reduce the need for travel and time off work and greater overall convenience compared to face-to-face medical consultation [29,30]. More recently, telehealth models have emerged at the forefront at the outset of the COVID-19 pandemic, allowing delivery of healthcare services while containing the transmission of infection [30,31]. Pharmacists have also effectively embraced telehealth to provide ongoing monitoring and management to patients with chronic conditions during the pandemic [32,33]. The present dynamics of global healthcare has enabled students to appreciate telehealth as the next frontier in healthcare delivery. As one of the students stated in the interview, telehealth could also offer flexibility in working schedules for healthcare providers, thus enhancing their work–life balance. This reflection on the perceived benefits of telehealth beyond what it is credited for thus far underpins the larger potentials of digital health in the near future. Moving forward, it would be vital for faculties to consider incorporating education and training of telehealth skills into the pharmacy curriculum by means such as virtual OSCEs to future-proof pharmacy graduates. Future research could explore whether medical educators could utilise this form of virtual assessment for rural and remote students who are on placements.

While the majority of examiners also viewed the virtual OSCE as a great platform to upskill students in telehealth delivery, some were of the opinion that face-to-face interaction with patients is still essential for students to develop soft skills such as empathy. The importance of empathy in patient care is unquestionable. Research indicates that empathy has a significantly positive impact on patients’ health outcomes [34,35]. Aside from increasing patient satisfaction and adherence, practitioner empathy also strengthens patient enablement [35]. However, with digitization of healthcare, there is a deep concern that interactions can become impersonal and expression of empathy is reduced [36].

As suggested by previous commentaries [4,5], it is challenging to capture non-verbal communication skills via video conferencing tools. In a study conducted at a tertiary hospital in Japan, comparing telemedicine consultation with face-to-face consultation among doctors and patients, the researchers reported that affective behaviour patterns, particularly empathy-utterances, were less evident in the telemedicine consultations [37]. Our study reinforces these views as both examiners and students mentioned difficulty in either expressing non-verbal communication, such as cues for empathy or giving eye contact, while examiners expressed the lack of ability to observe and assess students’ non-verbal communication. Even though these challenges were mainly due to the limitations of the online platforms, such as the inability to observe the full body or difficulties with audio, making it more difficult to hear, students showed self-awareness of these challenges and a desire to improve.

It is positive that students were able to acknowledge that providing patient care via telehealth can be a challenge and that it requires a different set of skills than counselling a patient in person. This was evident with our students’ motivation to practise virtually to mimic their actual assessments over Zoom. Additionally, faculty should consider targeted training focused on the development of virtual communication skills. Perhaps it is time for educators to consider integrating the construct of digital empathy in the pharmacy curriculum [36]. To this end, both students and examiners suggested that a combination of the traditional face-to-face and virtual OSCEs in the curriculum will be able to equip them holistically to render good patient and pharmaceutical care.

This study has its limitations. This study was only conducted within one pharmacy school in Melbourne, Australia. Although a high response rate was achieved, the findings might not be generalizable to other schools or countries, depending on the program and strategies in place to adapt during the pandemic. Nevertheless, this study adds to the qualitative findings from both students and examiners around their experiences of virtual OSCEs and provides insight into the potential usefulness of virtual OSCEs in future pharmacy programs.

## 5. Conclusions

The need for remote online delivery of assessments saw innovative ways of undertaking OSCEs. We found that the virtual OSCEs were an opportunity to mimic telehealth and current practice. While students and examiners embraced the virtual OSCE process, face-to-face OSCEs were still considered important and irreplaceable. This is most evident around aspects of the development of non-verbal communication skills, which was challenging to observe virtually. Future opportunities for OSCEs to be delivered both face-to-face and virtually should be considered.

## Figures and Tables

**Table 1:** Zoom OSCE process for each student [3].

Step	Description
1	Students dial into the “Zoom OSCE Lobby” Meeting link at their allocated time
2	Technology (audio/visual) and identification checks are conducted
3	Students are sent to their “Zoom OSCE Room” for their examination
4	OSCE interaction with the examiner occurs in the Zoom OSCE Room
5	Students leave the Zoom OSCE Room at the conclusion of their OSCE

**Table 2:** Online survey results (*n* = 167).

Item	Strongly Disagree/Disagree *n* (%)	Neither Agree/Disagree *n* (%)	Strongly Agree/Agree *n* (%)
I prefer virtual OSCEs compared to face-to-face OSCEs	38 (23%)	74 (44%)	55 (33%)
I feel more anxious during the virtual OSCE compared to face-to-face OSCE	79 (47%)	55 (33%)	33 (20%)
The virtual OSCE felt more challenging compared to face-to-face OSCE	63 (38%)	84 (50%)	20 (12%)

**Table 3:** Subthemes from Theme 1 and representative quotes.

Subthemes	Participant Group	Representative Quotes
1.1. Benefit of virtual OSCE at home	Students	*“More flexible, less stress when at home”—Online Survey*
		*“Well, for me, I travel, you know, if I had to come to university I would have to travel around an hour or so. So that you know if when you take that out of the picture I get that extra time to maybe prepare. And so that was, I really appreciated that from my perspective. I’ve been able to save up a lot of time because I don’t have to go to university.”*—*Interview Student 8*
		*“Virtual OSCE is pretty good because it saves a lot of time like on traffic. That’s one of the best parts.”*—*Interview Student 3*
		*“Convenient, just have to show up at the allocated time without worrying about public transport etc.”*—*Online Survey*
	Examiners	*“Flexibility I suppose, flexibility in terms of the fact that again, I was able to examine from the home, students were able to sit from their place of choosing, I was able to put in my background a nice pharmacy environment, which may be looked a bit more realistic than just being in a counselling suite. I think that was all a positive experience to come out of it.”*—*Interview Examiner 3*
1.2 Comfort	Students	*“I would say seamless online learning, it’s being able to conduct the OSCE in the comfort of your own home. I think you get to have that full sleep in. For me it’s to get to uni. It’s quite far. It’s about an hour and 40 or so minutes. So by the time I get there on public transport, and they give you can get quite fatigued and then having to be so I guess surrounded by friends can be a good thing… So I think doing it at home, you see people briefly get to be at home, get to sleep well and then go into your OSCE. I think it’s more comfortable in that sense.”*—*Interview Student 7*
1.3 Convenience	Students	*“How easy it is to access our notes and other resources.”*—*Online Survey*
	Examiners	*“Certainly, more convenience and I guess a better use of my time in that setting. I found it very easy to be having them on the screen there. And like I’ve got two screens here. So I think having two screens is a good advantage as opposed to just one. So having like the rubric open on one screen, and student on the other screen, I thought worked really well. And it was quite easy for me to do both simultaneously.”*—*Interview Examiner 2*
		*“But I did set up two computers and made sure that I actually connected through different internet connections, so that if one went down, you know…”*—*Interview Examiner 4*
		*“I think that flexibility and the, I guess also the ability to really have what you need on your screens, and I know some people have three screens. And so that perhaps as an examiner, you may appear to sort of be able to look at a few things without distracting as much because I guess if you’re looking at your notes, or trying to confirm something, in person, that’s really obvious…”*—*Interview Examiner 7*
1.4 Reliance on Technology	Students	*“I felt like with the technical, I understand like, it’s completely unavoidable in some cases as well. But it just threw me off a little bit. Because then I’m like, Am I missing my OSCE? Is the examiner waiting for me? But aside from that, it was just the whole… I was pretty comfortable during the OSCE.”*—*Interview Student 4*
		*“Convenient but then the internet connection was not always stable.”*—*Online Survey*
1.5 Flexibility	Examiners	*“I like the fact that, because it was conducted virtually, I have a bit of control over the timing. If the students came in, you know, a little bit later they were flustered, I could say you know calm down and then I can start the seven minutes. I always stuck to the 7 min allowance but it was good to have a little bit of flexibility even to say to the student before I can you just give me a minute to catch up on my marks with the previous students. So all in all, from my experience and I’m doing this, honestly, it was seamless.”*—*Interview Examiner 1*
1.6 Future practice	Students	*“And I definitely think we should have a combination of face to face and telehealth like communication, because like if someone’s working, you know in the city and they have to talk to someone who’s in a rural area. This is the best way of communication and like we’re gonna have to deal with technology, it’s like already prominent now.”*—*Interview Student 4*
		*“Zoom OSCEs are more convenient and perhaps more comfortable for me, but I think the face to face OSCEs can better prepare us for future practice.”*—*Online Survey*

**Table 4:** Subthemes from Theme 2 and representative quotes.

Subthemes	Participant Group	Representative Quotes
2.1 Sense of security	Students	*“I think it was good how, because I was at home, I was like, in a safe environment. Like there was no one else around me that stressed me out like it was just me. So I feel like it was less stressful than like being in a room with lots of people nervous waiting for it. Just like I feel like you just like sit and look at everyone’s faces and you’re like, oh, like they’re worried too. Like, should I be as worried as them kind of thing.”*—*Interview Student 1*
		*“I guess the best stuff is that I don’t have to talk with my peers before and after. Talking to them before, most of them are really nervous. And I’m kind of person to not put myself in a nervous state. But if people keep talking too much around me, I get nervous as well. And the best thing is after is that because after my test I don’t want to talk about it anymore. I just want to get rid of it. And if that’s OSCE during in school, I have to talk with my peers, Oh how did you go…yeah”*—*Interview Student 3*
		*“I did feel anxious for the virtual OSCE however it was to a lesser extent compared to the face to face. I think the environment of the real life OSCE makes me more anxious whereas with the virtual one it was more calm and collected. The virtual OSCE allowed me to mentally prepare myself without being surrounded by others”*—*Online Survey*
		*“Quick and easy process. Low stress environment because there weren’t other stressed students around.”*—*Online Survey*
2.2 Reduced nerves	Examiners	*“Well, the students mostly do struggle with communication… If anything, maybe some of the students were more confident on screen than they would have a person, well at least seems that way. And some get very nervous in person…. So that’s certainly a difference.”*—*Interview Examiner 4*
		*“But then I think of all the times when the poor things are so, so nervous. And what I feel more like is that I’m seeing them suffer, whereas, I don’t know whether they were more relaxed (virtually) or it’s just that, you know, you don’t see things like their hands actually shaking when it’s on video… but I suppose you don’t get the full feel of how they are feeling because you can’t see the whole body and you know, whether they’re absolutely shaking literally in their boots or not.”*—*Interview Examiner 8*
		*“I personally find it quite a positive experience… I thought it was a positive experience for me. It was more comfortable. Like, doing the timing itself on the phone…When you’re actually there physically in the OSCE is quite confronting, and quite like everything needs to happen so quickly. Whereas, when you’re there, on your own, and you’ve got the dual screens and you’ve got the capacity to do things. It becomes a lot easier.”*—*Interview Examiner 10*
	Students	*“But being at home, I think did help my nerves.”*—*Interview Student 8*
		*“I felt not as stressed as I would in normal face to face physical environment. Um, I had my phone with the timer on my desk as well. And I think that helped, like calm my nerves because I was like, I’m travelling like well for time as compared to the physical like environment where you don’t have a counter like, time. Usually the thing is the timing is the source of stress. But because I had like, I was able to look at it this time it was less stressful.”*—*Interview Student 2*
		*“So I think for some people, and like for me, in some ways that was a bit more better because I had time to relax in my own room. And, you know, have my own prep before, before going into the room, and also in some way that was a bit informal, which that was another aspect of it, but it was great like to see that whole lead up being avoided… So like before, I have an assessment, or maybe I like play my music and like I’ll nibble on something. And so I just like get myself in the zone”*—*Interview Student 4*
		*“Doing the OSCE in a familiar environment encouraged me to feel more relaxed and at ease”*—*Online Survey*

**Table 5:** Subthemes from Theme 3 and representative quotes.

Subthemes	Participant Group	Representative Quotes
3.1 Pharmacists in training	Students	*“This is also one of the great ways to learn as a pharmacist in training”*—*Online Survey*
		*“Although it was a different experience, I am glad that we were given a chance to do a virtual OSCE as in practice, there may be cases where we have to counsel online so it really was beneficial in preparing us for what to expect in the future”*—*Online Survey*
		*“It feels appropriate to the challenges of today*—*having to complete telehealth consultations”*—*Online Survey*
		*“We are in a, I guess, electronic technology age where we do depend on our phones, our laptops, the internet pretty much everything. And what I do see from the virtual OSCE is I do see pharmacists in the future, using technology, such as this to talk to patients who are unable to come into the pharmacy. So, maybe in a community, or maybe even in a hospital if let’s say, like there is with coronavirus, they want to limit the amount of people with each other. This covers how you know health professionals can get in contact with patients.”—Interview Student 6*
		*“I like that this is preparation for telehealth in my future career.”*—*Online Survey*
		*“I think that virtual OSCEs will really help in terms of the growing telehealth which is emerging especially in times like this with the Coronavirus and will allow me to practice communicating with patients especially if you are not talking to them directly.”*—*Online Survey*
3.2 Telehealth	Students	*“… since like now telehealth is such a big thing. I feel like there’s benefits face to face and by Zoom because if there are cases such like the Coronavirus, telehealth is so important. So we would have to learn how to communicate with other people online and still be able to give patient centred care, I guess, to the best of our abilities but just through a different medium. And I feel like having this opportunity to actually do that, has been really beneficial”*—*Interview Student 9*
		*“Doing the OSCE virtually was a great opportunity for me to learn more about Telehealth and it taught me how to be flexible. For example what to do if the patient can’t hear us, how to explain certain medications without having the patient physically in front.”*—*Online Survey*
		*“Telehealth is becoming far more common in modern day pharmacy and an OSCE that involves it would be very useful for developing online communication skills.”*—*Online Survey*
		*“In recent years telehealth has been coming especially due to the COVID-19 situation and the future I think may be a staple of pharmacy practice. In my own country, it is actually used pretty often. So for example, pharmacy technicians will be stationed at the pharmacy or pharmacists can be called in from a different location. So this will allow for more flexible workings and it’s a lot easier on the pharmacy side as well.”*—*Interview Student 5*
	Examiners	*“To prepare students for telehealth and it would be interesting actually to compare how the students, you know conduct themselves, and you know whether they meet the same criteria in the virtual versus non virtual world.”*—*Interview Examiner 1*
3.3 Hybrid OSCE for skill development	Examiners	*“If telehealth becomes like, you know, the normal for pharmacists, then that’s brilliant, because like, I think it’s a really great way to train them…I think, you know, for what we, you know, for the unprecedented sort of time that we have. The assessments were quite good. But I wouldn’t sort of think that they would be sufficient for the future training of other students. I think the face to face assessments are still superior. That’s just from just because some of them clearly are having sort of trouble talking to people in person already. And then online, that is a bit harder. So, because they are obviously quite young and have less life experience and things like that. So I think my experience overall with the virtual OSCEs, 100%, like I’m very happy, but in terms of helping out the students, I think, then it might not be as sufficient sort of way of training them and assessing them in the course overall.”—Interview Examiner 5*
		*“I think it’s something we could certainly build into the course and it’s a good one for like telehealth and all that kind of stuff. But I think it does take away from the fact that you do see a real person in front of you. So I still think face to face contact is essential to improve students’ empathy, and improve those kinds of skills.”*—*Interview Examiner 2*
	Students	*“So I think it’s a great concept. And I think it’s so relevant, but I’d like a mix of face to face.”*—*Interview Student 4*
		*“A mix it would be more preferable. So maybe alternating time because sometimes there is two OSCEs we have a period of time. So one physical one virtual, so to experience two parts of what could be possible in the future. So something more holistic in nature.”—Interview Student 5*

**Table 6:** Subthemes from Theme 4 and representative quotes.

Subthemes	Participant Group	Representative Quotes
4.1 Difficulty displaying good non-verbal skills	Students	*“When you’re doing online, like video, interviewing and things like that, it’s always harder to gauge people’s non-verbal cues. I think in person you can kind of sense when someone’s about to start talking, or how someone’s responding to what you’re saying. But when it’s online, I find it much more difficult…. I would pick traditional OSCEs for the human interaction. Because I think it’s, you’re better able to, I guess, show yourself and your energy and your personality and I guess non-verbal cues and things like that in person.”—Interview Student 7*
		*“You aren’t able to really see the bottom half, the hands, you’re only able to see the face, most of the time so without that, non-verbal communication has been cut off. But still I feel that even though just seeing the face, you are still able to get the information, or able to understand what you have said… But, obviously it’s not perfect.”*—*Interview Student 5*
		*“I don’t mind doing either as the process is still similar. However non-verbal communication may not be delivered as readily through the computer screen.”*—*Online Survey*
	Examiners	*“The face to face had a massive advantage in terms of being able to have that interpersonal you know, non-verbal communication skills showing off and that was quite difficult to do in the virtual OSCE. As obviously, but it’s, you know, to be expected. So it was a bit harder to… Yeah, just have that interpersonal skills coming out from the students, so it (was) harder to sort of connect with them as well. And when you’re playing the patient or you know, yeah, I thought that was the biggest, most noticeable effect of that.”*—*Interview Examiner 5*
		*“But the only thing I noticed was students were probably more reliant on resources around, because I’m sure the visual OSCE gave them more opportunities to have everything around them and so they were very concerned because you could see their eyes moving. In person you know they’d be very fixated on speaking to you, whereas in the virtual ones not everyone, but sometimes there would be looking elsewhere, and not so much, paying attention to the patient that was in front of them.”*—*Interview Examiner 9*
4.2 Empathy	Students	*“It was harder to understand the patient, and show empathy over a computer screen”*—*Online Survey*
		*“I guess compared to being a real patient, on screen does make a patient’s emotion less easier for me to observe. Their statement are quite straightforward though…. normally if we were sitting face to each other face to face, it’s easier to observe like the, like eye contact, like through the like tiny movement. Yeah.”—Interview Student 3*
		*“It’s different to when you’re in person. And I guess it doesn’t tone down how empathetic you are and I guess it just, it can’t… some people may come up as different if it’s in a camera or if it’s face to face.”*—*Interview Student 6*
	Examiners	*“The potential for lack of engagement that you might get, and that’s of empathy and non-verbal communication, those sort of aspects that you probably do pick up better in person”*—*Interview Examiner 4*
		*“The fact that I did notice that some of the poorer English language students were obviously reading off a script, which if you think about that from a real life perspective… Although they did look quite formulaic, and I had one student who she seriously sounded like a robot, I thought the way in which there was little emotion that she showed throughout the OSCE interview… The fact that she was so robotic, and maybe that’s that would have been the same from a face to face OSCE but it was clearly noticeable in the OSCE last week that was run* via *zoom. That formulaic nature and just showing very little emotion to any response that I gave, which was quite, just quite, quite concerning and disappointing, but again, I don’t know whether that same student would have performed the same way if it was a face to face OSCE.”*—*Interview Examiner 3*
		*“Trying to pick up on some of the students’ non-verbal language is maybe not quite as easy. I probably took a very, almost digital approach in that. You know, it’s hard with cameras because cameras can be at different points. And so you’re looking at the person, but, you know, they’re looking at their screen rather than the camera so they don’t always look like they’re making eye contact with you.”—Interview Examiner 8*
		*“I was more conscious that I needed to engage the student, which is kind of strange because I think for me, I find it quite strange because in person that happens naturally anyway because you’re in front of a physical person whereas this is behind the screen. And so I think perhaps I was more aware to, to be friendly and sort of use gestures and normally you can see body language really easily in front of the person but in this case you have a screen so I was making sure that, you know, if I was sneezing (for the case) or whatever it was and it was quite, quite obvious as well.”—Interview Examiner 9*

**Table 7:** Theme 5 and representative quotes.

Participant Group	Representative Quotes
Students	*“Yes, so me and a few of my peers, we decided to jump on to Zoom and then pretend that we were in the Zoom OSCEs. Not necessarily through the meeting format, but all the breakout room formats, but more so just some general meeting. And so we, one person would pretend to be the patient and then the other person would be unfamiliar with what the patient was presenting with. And then we just play it out within the seven minutes. And we take turns doing that…. we decided that because the OSCE was going to be done over zoom, we would jump over to zoom meetings and then try it out and put up our backgrounds and yeah, test everything out, test the waters before we started.”—Interview Student 7*
	*“Did practice on Zoom with my friend. And that helped a lot.”—Interview Student 3*
	*“I prepared for it a bit differently compared to the face to face OSCE that I prepared for last year… as you progress through university, your strategies do change over time. But in this case, what I did was, I did have zoom calls with my colleagues, and we practice the OSCE with each other. And I think that really helped me understand my tone of voice, how, how loud I should be speaking for the other person to be able to understand through the screen. I also wrote a script, which was sort of going which would help me organise my thoughts and the things that I’ll see.”—Interview Student 8*
	*“We went on a zoom call, not unlike this one. And we timed ourselves using our phones. And we came up with scenarios… So yes, I’m just trying to like reenact the environment of an OSCE”—Interview Student 2*

## Data Availability

Original data are stored by the authors. Participants did not consent to have their raw data made publicly available.
